# Draft Genome Sequence of Bordetella avium Nh1210, an Outbreak Strain of Lockjaw Syndrome

**DOI:** 10.1128/genomeA.00120-15

**Published:** 2015-03-12

**Authors:** Luisa Z. Moreno, Terezinha Knöbl, André A. Grespan, Maria R. Felizardo, Cleise R. Gomes, Thais S. P. Ferreira, Maria Gabriela Xavier de Oliveira, Livia Myriantheus, Andrea Micke Moreno

**Affiliations:** a School of Veterinary Medicine and Animal Science, University of São Paulo, São Paulo, Brazil; b Wildvet Clinica Veterinaria & Resort, São Paulo, Brazil

## Abstract

Bordetella avium is a highly contagious bacterium that infects the upper respiratory tract of birds. B. avium Nh1210 is an outbreak strain of lockjaw syndrome in juvenile cockatiel chicks (Nymphicus hollandicus). Here, we report the draft genome sequence of strain Nh1210.

## GENOME ANNOUNCEMENT

Bordetella avium is a highly contagious bacterium that infects the upper respiratory tracts of wild and domesticated birds. Commercially raised turkeys are particularly susceptible to bordetellosis (turkey coryza), leading to large economic losses due to a high predisposition to secondary infections. The infection in psittacine birds has been characterized by rhinitis, sinusitis, and temporomandibular joint rigidity (lockjaw syndrome) (1). The first outbreak of lockjaw syndrome in cockatiel chicks (Nymphicus hollandicus) was reported in 1994 in Canada (2). Since the emergence of reports of human disease associated with B. avium (3, 4) and the increase of cockatiel chicks as a pet bird, B. avium is characterized as an opportunistic pathogen that presents a risk to public health by its zoonotic potential.

The only published B. avium genome (accession no. NC_010645) is related to B. avium strain 197 N, isolated from a diseased turkey in the United States (5). It presents limited synteny and lower DNA and protein similarities than those of the other Bordetella genomes. Here, we present the draft genome of B. avium Nh1210, an outbreak strain causing lockjaw syndrome in juvenile cockatiel chicks (N. hollandicus). Strain Nh1210 was previously characterized as being highly virulent and having a limited resistance profile (6). Genomic DNA was purified with the illustra bacteria genomicPrep mini spin kit (GE Healthcare) and used for paired-end library preparation with the Nextera DNA sample prep kit (Illumina) and sequencing through the Illumina MiSeq platform.

Sequencing resulted in 573,016 paired-end quality-filtered reads (approximately 30-fold coverage). The *de novo* assembly was performed with the CLC Main Workbench 7.0 (CLC bio, Denmark) and resulted in 68 contigs, with an *N*_50_ of 118,218. The resulting contigs were ordered according to the B. avium 197 N reference genome. The B. avium Nh1210 draft genome presented 3,684,716 bp, with a G+C content of 61.5%. Automatic genome annotation was performed with the xBASE bacterial genome annotation service (7). In total, 3,430 coding genes, 61 tRNA genes, and 9 rRNA genes were identified. In addition, two prophages were detected using the PHAST (PHAge Search Tool) (8).

In regard to virulence factors, Nh1210 lacks the gene clusters encoding the pertussis toxin and adenylate cyclase components, as expected; however, it presents a 41% identical orthologue to the dermonecrotic toxin gene (*dnt*), two fimbrial loci, and >10 fimbrial subunits differing from the mammalian-adapted bordetellae. The B. avium Nh1210 genome differs from the reference genome 197N by presenting intact genes associated with virulence mechanisms of B. avium (three type IV pilus gene clusters and the *bvgS* gene), confirming that the *bvgS* C(4) tract strain is virulent, at least for cockatiel chicks. A comparative genomic analysis of the strains 197 N and NH1210 with other B. avium genomes will be reported in the future to enhance the study of host specificity factors for B. avium strains.

### Nucleotide sequence accession number.

This whole-genome shotgun project has been deposited in DDBJ/ENA/GenBank under the accession no. JWMK00000000.
